# Porencephaly and cortical dysplasia as cause of seizures in a dog

**DOI:** 10.1186/1746-6148-8-246

**Published:** 2012-12-27

**Authors:** Gisele Fabrino Machado, Maria-Gisela Laranjeira, Augusto Schweigert, Guilherme Dias de Melo

**Affiliations:** 1 Department of Animal Clinics, Surgery and Reproduction, UNESP - Univ Estadual Paulista, College of Veterinary Medicine, Rua Clóvis Pestana, 793, Araçatuba, SP, CEP 16050-680, Brazil

**Keywords:** Brain, Canine, Central nervous system diseases, Cerebellum, Hippocampus, Neuropathology

## Abstract

**Background:**

Seizures are a common problem in small animal neurology and it may be related to underlying diseases. Porencephaly is an extremely rare disorder, and in Veterinary Medicine it affects more often ruminants, with only few reports in dogs.

**Case presentation:**

A one-year-old intact male Shih-Tzu dog was referred to Veterinary University Hospital with history of abnormal gait and generalized tonic-clonic seizures. Signs included hypermetria, abnormal nystagmus and increased myotatic reflexes. At necropsy, during the brain analysis, a cleft was observed in the left parietal and occipital lobes, creating a communication between the subarachnoid space and the left lateral ventricle, consistent with porencephaly; and also a focal atrophy of the caudal paravermal and vermal portions of the cerebellum. Furthermore, the histological examination showed cortical and cerebellar neuronal dysplasia.

**Conclusions:**

Reports of seizures due to porencephaly are rare in dogs. In this case, the dog presented a group of brain abnormalities which *per se* or in assemblage could result in seizure manifestation.

## Background

Seizures are a common problem in small animal neurology and it may be related to underlying diseases. Brain anomaly associated with seizures was described in 4.16% of 240 dogs in a retrospective study [1].

Porencephaly is an extremely rare disorder of the central nervous system (CNS) involving a cavity filled with cerebrospinal fluid (CSF), in the brain's parenchyma, usually connecting the ventricles to the brain surface. The lesion is associated with ischemic or hemorrhagic episodes and is characterized by a cavity, or cavitations in brain tissue, of variable size and location, given different names according to presumed mechanism and morphology [2]. Extensive porencephaly communicates through the subarachnoid space to the ventricles, which shows similar morphological findings to those of open-lip schizencephaly. Patients with these two types of defective lesions present with severe developmental delays and intractable epilepsy [3]. Cavitation in schizencephaly is lined by dysplastic cortex, usually associated with polymicrogyria [4].  Comparing  with  hydranencephaly,  porencephaly describes less extensive defect in the cerebral walls, which may not communicate with the CSF compartments [5].

The main causes of porencephaly in humans seem to be vascular cerebral lesions caused by traumatism, infections or congenital defects [6,7]. In cases of congenital porencephaly, the cause can be genetic or due to perinatal vascular lesions [2,7]. In Veterinary Medicine, cases of porencephaly are infrequently described in cattle, sheep and goats, and they are usually related to viral infections such as Akabane virus, bovine viral diarrhea and ovine gammaherpesvirus [8-11]. In sheep, porencephaly and hydrocephaly may be related to copper deficiency [12].

There are few reports of porencephaly in dogs and cats. Mackillop [13] diagnosed a case by magnetic resonance imaging (MRI) in a Labrador Retriever dog, where the author suspected that the condition was secondary to a prenatal forebrain infarct. Other recent publications about CSF-filled cavities reported cases of porencephaly in dogs and cats also diagnosed by MRI [5,14]. This infrequent lesion should be considered as differential in cases of seizures, and therefore, the aim of this article is to describe the clinical and pathological aspects of a dog with porencephaly.

## Case presentation

A 1-year-old intact male Shit-Tzu dog was referred to the Veterinary Teaching Hospital of the College of Veterinary Medicine – São Paulo State University (UNESP) with a history of weakness in all four limbs and muscle atrophy, reluctance to move, ataxia and episodes of generalized tonic-clonic seizures. The clinical findings of the neurological examination are shown in Table 1. Differential diagnoses included cerebral lesions, infectious encephalitis, hydrocephaly and vestibulo-cerebellar syndrome, and to aid the differentiation, the cerebrospinal fluid was collected. However, during the post-anesthetic procedures, the dog had a cardiorespiratory arrest and died. 

**Table 1:** Neurological examination of the dog with porencephaly and focal cerebellar vermis atrophy

**Test**	**Response**
*Mentation*	depressed
*Motor coordination*	ataxia, forelimb hypermetria, weak gait
*Limb movement*	flaccid tetraparesis, hindlimbs more affected than forelimbs
*Myotatic reflex*	increased in all four limbs
*Muscle atrophy*	mild
*Proprioceptive positioning*	present in forelimbs, absent in hindlimbs
*Righting reaction*	normal
*Intention tremor*	present
*Nystagmus*	horizontal
*Pupillary light reflex*	normal, both direct and consensual
*Menace reflex*	absent
*Pain*	delayed response superficial pain, normal deep pain

**Figure 1:**
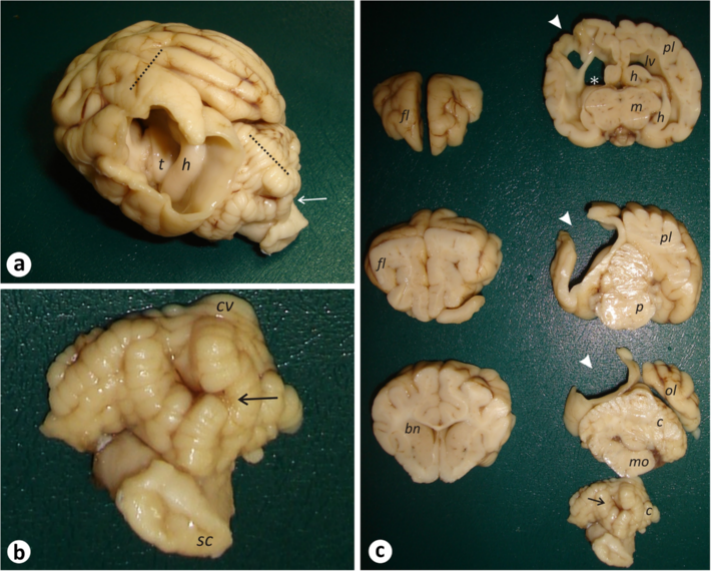
**Gross examination of the canine brain with porencephaly and focal cerebellar vermis atrophy. **(**a**) Brain: cerebral cleft in the left parietal and occipital lobes, creating a communication between the subarachnoid space and the left lateral ventricle. The dotted lines represent the point from where the sections destined to histopathology were taken. (**b**) Cerebellum: caudal view of the rhombencephalon exhibiting a focal atrophy in the caudal paravermal and vermal portions of the cerebellum (arrow). (**c**) Brain: transverse sections of the whole brain. Note the areas affected by the cleft (arrowhead) and also a thinning of the left hippocampal formation (*). Observe also a focal atrophy in the caudal paravermal and vermal portions of the cerebellum (arrow) *bn*: basal nuclei; *c*: cerebellum; *cv*: cerebellar vermis; *fl*: frontal lobe; *h*: hippocampal formation; *lv*: lateral ventricle; *m*: midbrain; *mo*: medulla oblongata; *ol*: occipital lobe; *p*: pons; *pl*: parietal lobe; *sc*: spinal cord; *t*: thalamus.

The cerebrospinal fluid analysis revealed no signs of alteration. Among the gross abnormality detected on post-mortem examination there was a cerebral cleft measuring approximately 1.8x1.0 cm at the left parietal and occipital lobes, creating a communication between the subarachnoid space and the left lateral ventricle. There was noticed also a focal atrophy in the caudal paravermal and vermal portions of the cerebellum and atrophy of the left hippocampal structure (Figure 1). The brain was fixed in 10% neutral buffered formalin and successive transverse sections (0.5 cm thickness) were made, embedded in paraffin, sectioned (3–4 μm) and stained with haematoxylin and eosin (HE). The brain from a 1-year-old dog which death was not related to neurological involvement was used as control. 

**Figure 2:**
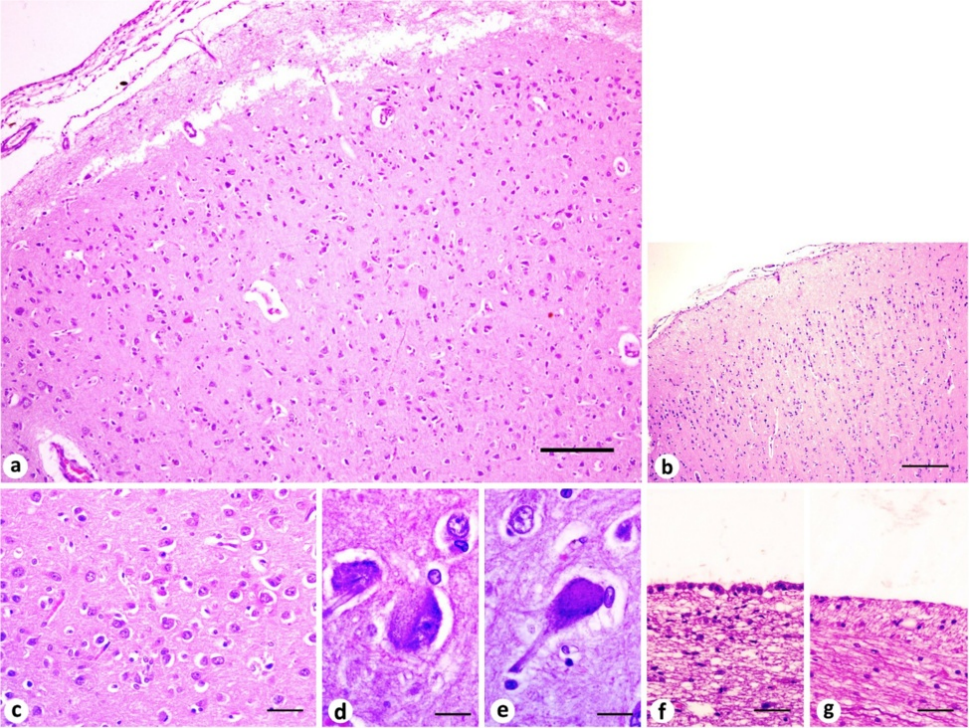
**Histopathological features of the cerebrum of a dog with porencephaly and focal cerebellar vermis atrophy. **(**a**) Parietal cortex: loss of normal cortical lamination, neurons in a disorganized arrangement. (**b**) Parietal cortex, control dog: well oriented neurons presenting a columnar organization. (**c**) Parietal cortex: neurons with aberrant size, disoriented position and arranged in clusters. (**d**) Parietal cortex: group of chromatolytic neurons. (**e**) Parietal cortex: large dilated degenerated neuron. (**f-g**) Lateral ventricle: Aspect of the ependymal lining intermittently with cuboid ciliated cells (**f**) or few pavimentous cells. (**g**) Hematoxylin and eosin. Scale bar = 200 μm (**a**, **b**), 50 μm (**c**, **f**, **g**), 20 μm (**d**, **e**).

**Figure 3:**
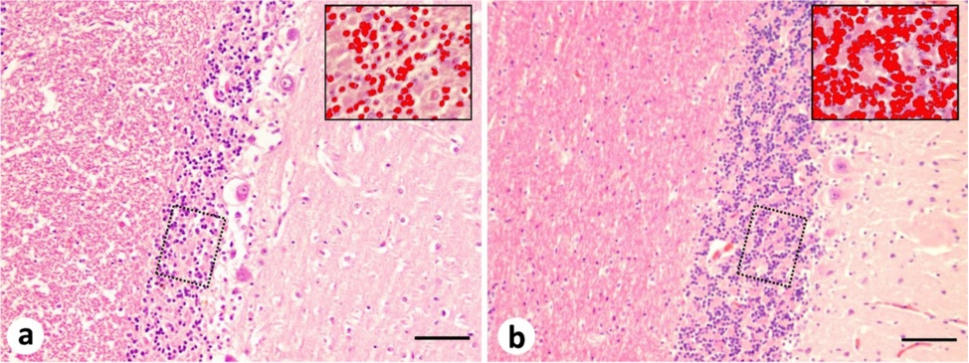
**Histopathological features of the cerebellum of a dog with porencephaly and focal cerebellar vermis atrophy.** (**a**) Low cellularity of the granule cell layer. *Inset:* neuronal density measurement in the cerebellar granular layer (nuclei in red). (**b**) Control dog: observe the normal cell density of the granule cell layer. *Inset:* neuronal density measurement in the cerebellar granular layer (nuclei in red). Hematoxylin and eosin. Scale bar = 100 μm.

At histological examination, in cerebral cortices, it was noticed a marked loss of normal cortical lamination (Figure 2a) when compared with the control cerebrum (Figure 2b), disorganized neurons with different sizes and shapes, arranged in clusters (Figure 2c). There was a high amount of abnormal neurons surrounded by reactive glial cells with focal distribution, pyramidal neurons with aberrant size, anomaly large, and also clusters of disoriented cells containing some chromatolytic (Figure 2d) and degenerated neurons (Figure 2e). Further, the ependymal lining of the lateral ventricles presented an irregular aspect, intermittently as a layer of cuboid ciliated cells (Figure 2f), or as few pavimentous cells (Figure 2g). The choroid plexus and the meninges presented with no alterations. In the cerebellum, the granular layer presented low cellularity (Figure 3a) but the Purkinje and the molecular layers showed no significant alteration. It was evident the hypocellularity in the granular layer when measured against the control cerebellum (Figure 3b). Both cerebella were subjected to a computerized image analysis (Image-Pro Plus 6.0; Media Cybernetics) to assess the area occupied by nuclei (in red) in a total area of 24,480 μm^2^: the dog with porencephaly and atrophy in the caudal cerebellar vermis presented 20.3% of nuclear area (4,978.5 μm^2^); and the control healthy dog presented 49.2% of nuclear area (12,035,5 μm^2^). Moreover, a marked increase in astrocytes cellularity was noticed in periventricular white matter, and in cortical areas the astrocytes were almost absent and showed an irregular disposition adjacent to neuronal clusters, in comparison with the healthy dog (data not shown). 

## Discussion

Porencephaly is an uncommon cerebral disorder in animals with almost nonexistent reports in dogs [5,13]. Seizures have been documented in animals with congenital abnormalities such as hydrocephaly, lissencephaly, and porencephaly [5,15].

In dogs, brain anomalies could be related to seizures in a low percentage of cases (4.16%) [1], however there are only few previous reports of seizures associated with porencephaly in this species [5]. On the other hand, seizures as consequence of idiopathic epilepsy are the most common brain disease in dogs [16]. Furthermore, reports of epilepsy due to focal cortical dysplasia in animals, as well as descriptions of neuronal malformation, are rare [17,18]. Although there is not a clear correlation between porencephaly and seizures, authors reported that porencephaly is often accompanied by amygdalar-hippocampal atrophy, which is usually related to the occurrence of seizures [19]. Hippocampal atrophy was also noticed in the case reported herein.

The main clinical manifestation of porencephaly is the occurrence of seizures [5,14], whereas the other clinical signs that the dog presented with, such as ataxia, dysmetria, intention tremors are related to vestibulo-cerebellar abnormalities [20,21]. Further association between vestibulo-cerebellar signs and porencephaly was observed in dogs and cats by Schmidt et al. [14], but without any noticeable cerebellar lesion. In the case reported herein it was detected a focal cerebellar vermis atrophy as well as low cellularity of the granule cell layer, which may be associated to the vestibulo-cerebellar signs, since cerebellar abnormalities associated with ataxia are frequently described in dogs, including degeneration, hypoplasia and localized defects [20-25].

In this case, CSF evaluation was normal. The dog had anesthetic complications without any apparent cause and died, and according to Gaynor et al. [26], it is a rare condition that might be associated with hypotension or cardiac dysrhythmias. The generic clinical signs render difficult the diagnosis of porencephaly; for humans, there is the possibility of mutation identification by molecular research [27], however, this is currently not applicable for animals.

The major gross alteration presented herein is consistent with porencephaly. We also found cerebral neuronal dysplasia and cerebellar vermis focal atrophy. Even though porencephaly has been associated with viral infections and nutritional deficit in ruminants and vascular disturbs in humans, the specific cause in dogs remains undetermined [7,15]. Viral infection is thought to affect endothelial cells during pregnancy and consequently causes vascular lesion and extensive loss of brain tissue, leading to the formation of cavitations [5,9,10]. Furthermore, the paucity of case reports make impossible to attribute this condition to a genetic predisposition of a specific breed. Consequently, since no cause could be determined, we describe herein a case of encephaloclastic (destructive) porencephaly, as suggested by Schmidt et al. [14].

## Conclusion

Porencephaly is an extremely rare disorder of the brain, with no previous description in Shih-Tzu dogs, and this report gives additional evidences to relate porencephaly to the occurrence of seizures. Therefore, as observed by Davies et al. [5] as well as in the case described herein, porencephaly must be considered as a differential diagnosis when associated to seizures. In this particular case, the additional clinical findings detected could be related to a vestibulo-cerebellar lesion, with no correlation to porencephaly. Consequently, we described herein a case of encephaloclastic porencephaly, neuronal dysplasia and a focal atrophy in the caudal cerebellar vermis, affecting the same animal and promoting the manifestation of a particular clinical condition.

## Consent

Orally informed consent was obtained from the owner of the dog for publication of this case report.

## Competing interests

None of the authors of this paper have any financial or personal relationship with other people or organizations that could inappropriately influence or bias the content of this paper.

## Authors’ contribution

GFM is the supervisor responsible for the case report and was responsible for collecting samples and interpretation of the histological findings. MGL participated in clinical research. AS and GDM performed the analysis and interpretation of the histological findings and photographed the images. All the authors helped to draft the manuscript. All authors read and approved the final manuscript.
